# Editorial: The power of relationships in human development: from prenatal bonding to attachment across the lifespan

**DOI:** 10.3389/fpsyg.2026.1955746

**Published:** 2026-09-17

**Authors:** Valentina Lucia La Rosa, Dario Alparone, Elena Commodari

**Affiliations:** 1 Department of Educational Sciences, University of Catania, Catania, Italy; 2 Department of Psychology, Faculty of Letters and Human Sciences, University of Western Brittany, Brest, France

**Keywords:** attachment, human development, interpersonal relationships, lifespan, prenatal bonding

Human development is shaped by relationships from before birth and throughout old age. Bowlby (1969) proposed that infants form a biologically based bond with their primary caregivers, one that serves a protective function and organizes how the child comes to expect closeness, comfort, and safety from others. Ainsworth et al. (1978) gave this proposal an empirical foundation through the Strange Situation procedure, which made it possible to observe and classify individual differences in the security of this early bond, and introduced the related concept of the secure base, the caregiver as a point of safety from which the child can explore the world.

Attachment theory did not remain confined to infancy for long. Hazan and Shaver (1987) extended the framework to adult romantic relationships, showing that the same patterns of security and insecurity identified in infants also organize how adults experience closeness, trust, and separation in couple relationships. In the decades since, the field has moved toward a genuine lifespan perspective, as reflected in comprehensive syntheses such as the *Handbook of Attachment* (Cassidy and Shaver, 2008) and *Attachment in Adulthood* (Mikulincer and Shaver, 2016), which trace how attachment processes continue to shape emotion regulation, caregiving, and psychopathology well beyond childhood. Attachment research has nonetheless remained concentrated on infancy and early childhood in practice, leaving the later stages of the lifespan comparatively underexplored. This Research Topic set out to broaden that scope. The 21 contributions gathered here span perinatal psychology, developmental and educational psychology, personality and social psychology, and clinical practice, and they trace the influence of relationships from the earliest prenatal interactions to romantic partnerships, family transitions, and bereavement in adulthood. Together, they suggest that attachment functions less as a trait fixed once in infancy and more as a process that continues to develop through the people who surround us at each stage of life.

## 1 The relational origins of development: prenatal bonding and the first year of life

Several contributions anchor this Research Topic in the earliest stages of the caregiving relationship. Cervellione et al. conducted a systematic review of the associations between prenatal bonding and emotion regulation in infancy and toddlerhood, showing that the affective tie a mother develops with her unborn child already lays groundwork for the child's later capacity to manage emotional states. Additionally, they identified psychological mediators and contextual moderators that shape this pathway. Building on this early window, La Rosa et al. examined psychological and social predictors of mother-infant bonding in a large sample of mothers followed through the first postpartum year, finding that postpartum depression, poor sleep quality, and low social and partner support were associated with weaker bonding, while adequate support from a partner emerged as a protective factor. Kurbatfinski et al. extended this line of inquiry across generations, showing that mothers' own adverse childhood experiences were linked, through their adult attachment pattern, to their preschool children's internalizing and externalizing behavior problems. Read together, these three studies point to a consistent pattern. The quality of the earliest bond is shaped jointly by the mother's own relational history, her mental health, and the support she receives from her environment, with measurable consequences for the child's subsequent development.

## 2 Attachment and socioemotional growth in early childhood

A second cluster of studies moves into early childhood and examines how relational experiences within and beyond the family support the growth of emotional and social competence. Ye et al. investigated sibling empathy among Chinese preschoolers, showing that empathic responsiveness between brothers and sisters is linked to general empathy and to the quality of the sibling relationship itself, a finding of particular relevance in a cultural context reshaped by changing family structures. Using a very different methodology, Oǧuz and Pinar offered a fine-grained qualitative case study of a 5-year-old girl's transition into kindergarten, illustrating through 9 months of naturalistic observation how expressive language and emotion regulation develop hand in hand as children navigate new social settings. Widening the lens to intergenerational relationships, Stephenson and Carstensen showed that grandparental support during childhood is associated with better emotional wellbeing in emerging adulthood, particularly among young adults who felt closest to their grandparents. This cluster reminds us that the relational network relevant to early development extends well beyond the parent–child dyad to include siblings, teachers, and grandparents.

## 3 Parent and peer attachment across adolescence

Adolescence emerged as the developmental period most densely represented in this Research Topic, and the contributions collectively depict it as a phase in which parental and peer attachment continue to exert a powerful, if increasingly complex, influence. Using a person-centered approach, Yüksel Doǧan and Demircioǧlu identified seven latent profiles of parent and peer attachment among more than two thousand Turkish adolescents, showing that these profiles differed systematically in life satisfaction, resilience, and basic psychological needs. Complementing this variable-centered picture, Li X. et al. found that parental autonomy support fosters adolescents' future orientation through a chain of growth mindset and hope, an effect that was further strengthened by high-quality peer relationships. Two studies turned to more strained family dynamics. Wei and Mei showed that parental psychological control is associated with heightened social anxiety among Chinese college students through reduced trait mindfulness and increased rejection sensitivity, while Cao et al. showed that general attachment predicts adolescent behavioral problems through distinct pathways involving parent–child attachment and self-control. Çiçek et al. added a protective perspective, showing that secure attachment is linked to greater family cohesion and lower peer bullying through the mediating role of positive childhood experiences. Sox et al. contributed a conceptual model that integrates Bronfenbrenner's ecological systems theory with attachment theory to explain how social support can buffer the effects of adversity and disrupted attachment on trauma symptoms in youth. Turning to a clinical population, Özaslan et al. examined how Attention-Deficit/Hyperactivity Disorder (ADHD) diagnosis, pharmacological treatment, and Internet addiction relate to the quality of parent–adolescent relationships, offering evidence directly relevant to families managing neurodevelopmental conditions. Terrell et al. showed that the quality of the parent–adolescent relationship indirectly shapes adolescents' conscious bias toward socioeconomically or ethnically marginalized peers through its effect on psychosocial wellbeing, while Talton et al. found that secure attachment to both parents buffers Black youth against the psychological toll of ethnical microaggressions, thereby reducing their reliance on emotional suppression as a coping strategy. Across this cluster, a coherent picture emerges in which parental and peer attachment operate not as isolated influences but as interacting systems that shape adolescents' emotional regulation, social cognition, and vulnerability or resilience to adversity.

## 4 Romantic attachment and couple functioning in adulthood

Three contributions extend the reach of attachment theory into adult romantic life. Schulz et al. used network analysis to map the personality correlates of relationship satisfaction, finding that insecure attachment, trust, mutuality, and sexual satisfaction each carried a unique association with how satisfied partners felt in their relationship. Brauer and Proyer examined how playfulness relates to attachment style and jealousy among single adults, replicating in this understudied population earlier findings from couples and showing that other-directed playfulness is associated with lower attachment avoidance. Finally, Marici proposed a new practice-based theoretical framework, the Love and Rejection Messages Theory, developed from more than a decade of clinical work with couples in crisis, offering clinicians a conceptual tool for understanding how everyday communicative exchanges shape the emotional climate of romantic relationships. These three studies, spanning quantitative, comparative, and theoretical approaches, converge on the idea that attachment security continues to organize how adults experience closeness, playfulness, and conflict in their romantic lives.

## 5 Attachment, family transitions, and the challenges of loss

Two further contributions illustrate how attachment processes intersect with major life transitions and losses. Li G.-Y. et al. showed that parent–child attachment is associated with young adults' intentions to have children of their own, an association mediated by family resilience and moderated by gender, a finding with clear relevance for research on fertility decisions in contemporary societies. At the other end of the emotional spectrum, Lenzo et al. investigated bereavement following oncological or traumatic loss, showing that psychosomatic dysregulation mediates the relationship between insecure attachment and the severity of prolonged grief symptoms. Together, these studies show that the legacy of early attachment experiences continues to inform how adults approach both the creation of new family bonds and the loss of existing ones.

## 6 Toward an integrated view across the lifespan

Closing the Research Topic, Ho and Rahaman conducted a systematic review and meta-analytic synthesis of the literature on attachment and relational quality published between 2014 and 2024, tracing continuities and discontinuities in how relationships shape developmental outcomes from the prenatal period to old age. Their synthesis offers a fitting frame for the Research Topic as a whole. It suggests that while the specific relational partners and challenges change across the lifespan, from caregivers in infancy to romantic partners and, eventually, the experience of loss in later life, the mechanisms linking relational security to psychological wellbeing remain broadly consistent.

## 7 Conclusion

The studies collected in this Research Topic point to relationships as a core, active influence on human development at every stage, rather than a fixed background condition. They also point to several directions for future research. Longitudinal designs that follow the same individuals from the prenatal period into adulthood remain rare and would allow a more direct test of the continuities suggested by this Research Topic. Greater attention to cultural and contextual variation, already visible in several of these contributions, will be important for avoiding overgeneralization from the findings obtained in specific populations. Finally, the studies addressing clinical populations and applied interventions point to a promising direction for translating attachment research into practice in pediatric, educational, or couple therapy settings. We hope that this Research Topic will be useful to researchers and practitioners alike, and we thank all the authors and reviewers who contributed their expertise to this Research Topic.
